# Mortality risk of Severe Acute Respiratory Syndrome cases classified as COVID-19: A longitudinal study

**DOI:** 10.1371/journal.pone.0309413

**Published:** 2024-08-30

**Authors:** Nádia Cristina Pinheiro Rodrigues, Joaquim Teixeira-Netto, Denise Leite Maia Monteiro, Mônica Kramer de Noronha Andrade

**Affiliations:** 1 Escola Nacional de Saúde Pública Sérgio Arouca/Fundação Oswaldo Cruz, Rio de Janeiro, Brazil; 2 Instituto de Medicina Social Hesio Cordeiro/Universidade do Estado do Rio de Janeiro, Rio de Janeiro, Brazil; 3 Faculdade de Ciências Médicas/Universidade do Estado do Rio de Janeiro, Rio de Janeiro, Brazil; 4 Universidade Federal do Rio de Janeiro, Rio de Janeiro, Brazil; Universidade Federal do Rio Grande do Norte, BRAZIL

## Abstract

**Background:**

The COVID-19 pandemic has significantly impacted global health, with diverse factors influencing the risk of death among reported cases. This study mainly analyzes the main characteristics that have contributed to the increase or decrease in the risk of death among Severe Acute Respiratory Syndrome (SARS) cases classified as COVID-19 reported in southeast Brazil from 2020 to 2023.

**Methods:**

This cohort study utilized COVID-19 notification data from the Sistema de Vigilância Epidemiológica (SIVEP) information system in the southeast region of Brazil from 2020 to 2023. Data included demographics, comorbidities, vaccination status, residence area, and survival outcomes. Classical Cox, Cox mixed effects, Prentice, Williams & Peterson (PWP), and PWP fragility models were used to assess the risk of dying over time.

**Results:**

Across 987,534 cases, 956,961 hospitalizations, and 330,343 deaths were recorded over the period. Mortality peaked in 2021. The elderly, males, black individuals, lower-educated, and urban residents faced elevated risks. Vaccination reduced death risk by around 20% and 13% in 2021 and 2022, respectively. Hospitalized individuals had lower death risks, while comorbidities increased risks by 20–26%.

**Conclusion:**

The study identified demographic and comorbidity factors influencing COVID-19 mortality. Rio de Janeiro exhibited the highest risk, while São Paulo had the lowest. Vaccination significantly reduces death risk. Findings contribute to understanding regional mortality variations and guide public health policies, emphasizing the importance of targeted interventions for vulnerable groups.

## Background

The COVID-19 pandemic has had a profound impact on global health, with various factors influencing the risk of death among reported cases.

Many risk factors have been reported to aggravate COVID-19, including advanced age, male gender, ethnicity, socioeconomic factors, geographic differences, quality of healthcare, underlying comorbidities such as hypertension, diabetes, obesity, chronic lung diseases, heart, liver and kidney diseases, tumors, immunodeficiencies, among others [1]. The occurrence of a cytokine storm has been suggested as an indication of the worsening of the disease [2, 3].

Scientific literature reports that most vaccines likely reduce symptomatic cases of COVID-19 [4] and its mortality [5]. However, several studies point to the adverse effects of vaccines against COVID-19 [6–9].

A greater understanding of the factors that affect the risk of death from COVID-19 can guide national public health policies. We chose the southeast region to carry out this investigation, as it is the most populous region in the country and covers important cities, such as São Paulo and Rio de Janeiro. This study mainly analyzes the main characteristics that have contributed to the increase or decrease in the risk of death among Severe Acute Respiratory Syndrome (SARS) cases classified as COVID-19 reported in southeast Brazil from 2020 to 2023.

## Methods

This is a cohort study using COVID-19 notification data obtained from the Sistema de Vigilância Epidemiológica (SIVEP) information system [10] from 2020 to 2023 in the southeast region of Brazil.

The SIVEP data includes all reported cases of SARS in the country. For this study, we selected only cases classified as COVID-19 in the southeast region.

The southeastern region of Brazil covers 42% of the Brazilian population according to the 2022 census [11] and includes some of the country’s main cities, such as São Paulo and Rio de Janeiro. It is divided into four states, the most and least populous being São Paulo and Espírito Santo.

All cases in which the first symptoms, hospitalization, and death occurred within the period from 2020 to 2023 were included in this study. Survival and risk of death over time were calculated within 90 days after the first symptoms.

We collected data about the state of residence, municipality of residence, date of first symptoms, date of hospitalization, date of death, age, the elderly population (≥65 years), sex, race (white, black, yellow, brown and indigenous), years of study (zero, 5, 9, 12, >12 and the “does not apply” category for individuals under 7 years old), type of residence area (urban, rural or peri-urban), epidemiological week, hospitalization (occurrence: yes/no), vaccination against COVID-19 (at least one dose), and presence of any risk factor for COVID-19 (postpartum woman, chronic cardiovascular disease, chronic hematological disease, Down Syndrome, chronic liver disease, asthma or another chronic lung disease, diabetes mellitus, disease chronic neurological disease, immunodeficiency or immunosuppression, chronic kidney disease, obesity, and others).

Relative and absolute frequencies were calculated for the predictors according to death status, as well as the means and standard deviations of age. Statistical tests were performed to compare the groups. Survival tables and Kaplan Meier curves were constructed to compare the survival experience in the different states, years, educational levels, races, sexes, and age groups.

Classical and Cox mixed-effects models were fitted to analyze the risk of death over time. The specification of the models is detailed below.

The classic Cox-adjusted model included the following predictors: age, sex, race, years of education, state of residence, zone of residence, year of first symptoms, hospitalization, and risk factors. Considering the presence of an interaction between COVID-19 vaccination and the year of first symptoms, three more models were built for each year in which vaccination was carried out in Brazil (2021 to 2023). These models excluded the variable “year of first symptoms” and included the variable “COVID-19 immunization”. We extracted only the adjusted Hazard ratios of vaccination concerning death to show in the table.

The first adjusted Cox mixed effects model included fixed effects of age, sex, race, years of study, state of residence, area, year of first symptoms, hospitalization, and risk factor; and the random effect of the municipality of residence (intercept). The second adjusted Cox mixed effects model included fixed effects of age, sex, race, years of study, area, hospitalization, and risk factor; and the random effects: of state of residence (intercept) and year of first symptoms (slope).

Prentice, Williams & Peterson (PWP) and PWP fragility models were fitted to analyze the risk ratio of the transitioning between an individual’s conditions (hospitalization and death) over time. PWP is a structured model for structured ordered events. In this model, it is considered that the baseline risk changes as the individual suffers the next event, that is, the risk of death depends on the previous hospitalization event. The frailty model includes random effects based on the classic Cox model. This causes individuals with smaller random effects (fragility) to experience longer times until the event occurs, and vice versa. The specification of the models is detailed below.

The PWP-adjusted model included the following predictors: age, sex, race, years of study, state of residence, area, year of first symptoms, and risk factors. Three other models were built for 2021 to 2023. These models also excluded the variable “year of first symptoms” and included the variable “COVID-19 immunization”. We extracted only the adjusted Hazard ratios of vaccination concerning death to show in the table.

The PWP fragility-adjusted model included the following predictors: age, sex, race, years of study, area, year of first symptoms, risk factors, and the random effect of the state of the residence. Three other models were built for 2021 to 2023 excluding the variable “year of first symptoms” and including the variable “COVID-19 immunization”. We extracted only the adjusted Hazard ratios of vaccination concerning death to show in the table.

We chose to use the PWP model due to the dependence between the hospitalization and death events. We chose to combine the PWP model into a fragility model to also access the random effect of the state of the residence.

Model goodness-of-fit measures were calculated for all models.

Graphs were used to illustrate the analyses. The R-project software version 4.3.2 was used in the analyses.

## Results

Across 987,534 cases, 956,961 hospitalizations, and 330,343 deaths were recorded over the 2020–2023. In 2020, 2021, 2022, and 2023, there were 317,009, 543,382, 103,952, and 23,191 cases of COVID-19, respectively. Of that, 107,323, 187,166, 31,244, and 4,610 died in 2020, 2021, 2022, and 2023, respectively (Table 1).

**Table 1:** Distribution of predictors according to COVID-19 death status in the southeast region of Brazil, 2020–2023.

*Predictors*	*Deaths (%)*	*P-value*
**Elderly**		0.0001
Yes	198956 (48.70)	
No	131387 (22.70)	
**Sex—*n (%)***		0.0001
Female	148000 (33.10)	
Male	182316 (33.70)	
**Race**		0.0001
White	170411 (34.80)	
Black	21734 (40.90)	
Yellow	3278 (32.10)	
Brown	87409 (34.20)	
Indian	155 (28.40)	
**Years of study**		0.0001
Zero	9483 (48.10)	
Five	46047 (46.90)	
Nine	27059 (41.90)	
Twelve	37805 (31.60)	
More than twelve	15661 (27.90)	
Not applicable	321 (5.20)	
**State**		0.0001
Espírito Santo	8303 (58.70)	
Minas Gerais	65624 (32.10)	
Rio de Janeiro	76460 (44.90)	
São Paulo	179956 (30.10)	
**Area**		0.0001
Urban	286945 (33.30)	
Rural	7611 (37.20)	
Peri-urban	1005 (35.70)	
**Year**		0.0001
2020	107323 (33.90)	
2021	187166 (34.40)	
2022	31244 (30.10)	
2023	4610 (19.90)	
**Immunization for COVID-19**		0.0001
Yes	71816 (32.10)	
No	258527 (33.90)	
**Hospitalization**		0.0001
Yes	311944 (32.60)	
No	8184 (59.40)	
**Risk factor**		0.0001
Yes	240311 (38.90)	
No	90032 (24.30)	

Year = first symptoms year; Not applicable = age < 15 years.

**Fig 1:**
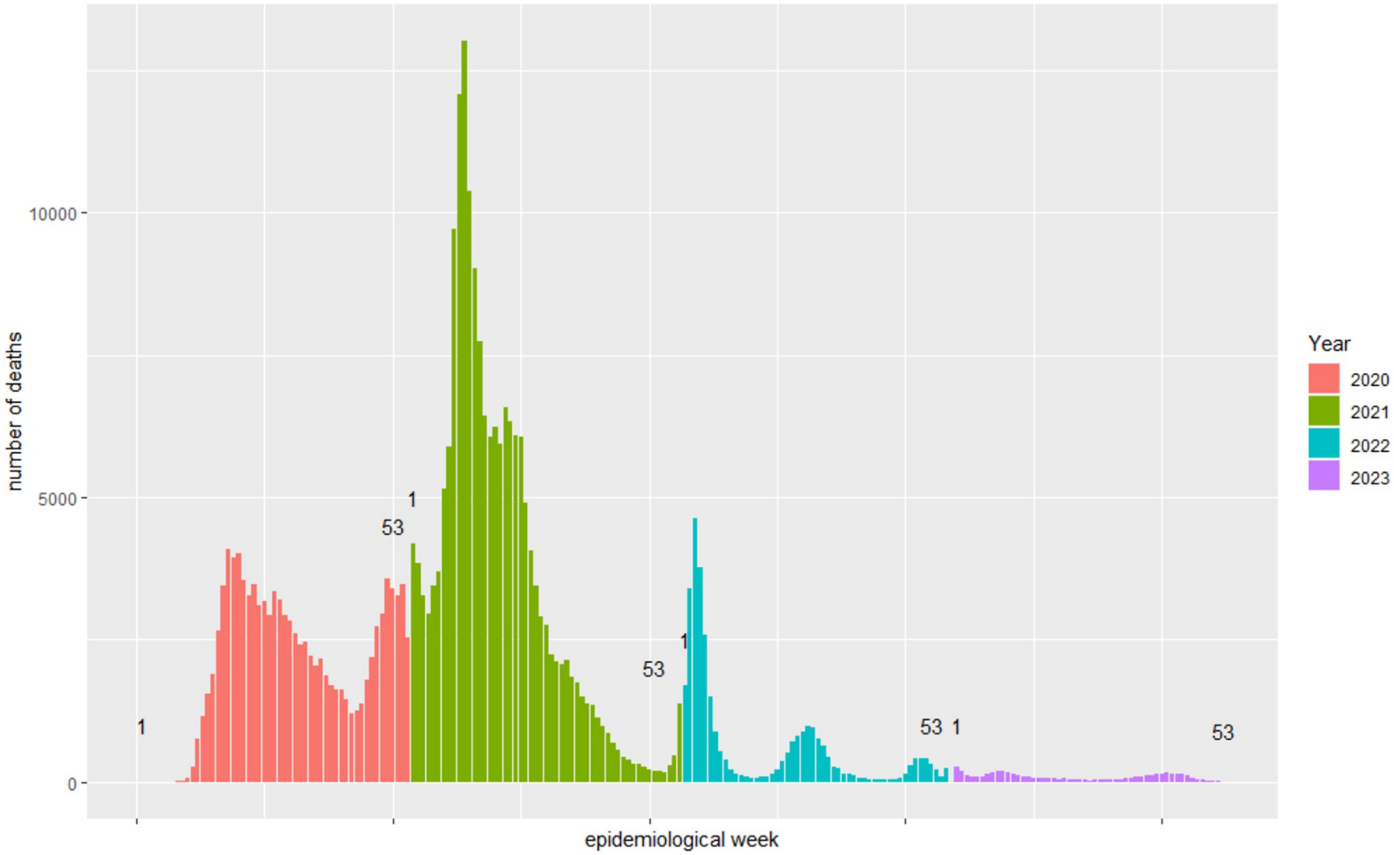
Distribution of the number of deaths of SARS cases classified as COVID-19 according to epidemiological week in the southeast region of Brazil.

The highest number of deaths occurred in 2021. From 2020 to 2022, mortality peaks occurred until the 18^th^ epidemiological week (2020: 4,089 deaths in the 18^th^ epidemiological week; 2021: 13,013 deaths in the 11^th^ epidemiological week; 2022: 4,621 deaths in the 3^rd^ epidemiological week (Fig 1).

The percentage of deaths doubled among the elderly. The average age of cases and non-cases of death were 67.38 (standard deviation (SD) = 15.92) and 54.90 (SD = 19.04), respectively. The death percentages were similar between men and women. Black individuals had the highest death rates. The higher the level of education, the lower the risk of death. The state of Espírito Santo had the highest percentage of deaths. The percentage of deaths in urban areas was slightly lower than in rural and peri-urban areas. A marked reduction in deaths is observed in 2023 (Table 1).

The death percentages were slightly lower for those vaccinated, but higher for those not hospitalized and those who had risk factors related to COVID-19 (Table 1).

**Fig 2:**
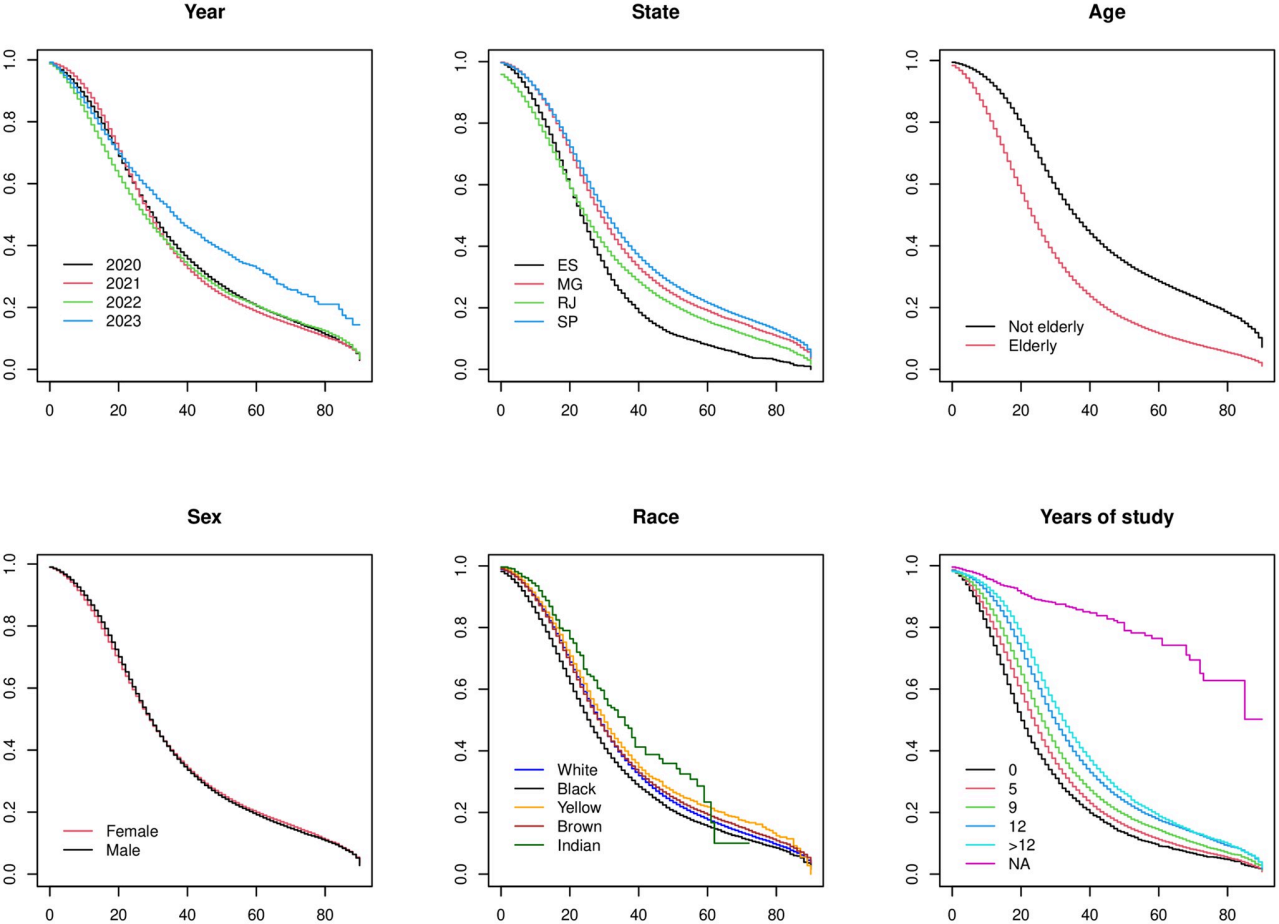
Survival of SARS cases classified as COVID-19 in the southeast region of Brazil, 2020–2023. Kaplan Meier survival analysis; Horizontal axis = time in days until death occurs; ES = Espírito Santo State; MG = Minas Gerais State; RJ = Rio de Janeiro State; NA = Not applicable (age < 15 years).

The survival rate was worse in 2021 (especially after 40 days of the first symptoms); in the state of Espírito Santo (especially 30 days after the first symptoms); in the elderly; in black individuals; and for those without education. Survival was similar for men and women (Fig 2).

The adjusted analysis indicates an increase of 2 to 3% in the risk of death for each year of life; a risk 7% higher in men; 11% higher in black people than in white; a reduction in the risk of death the higher the level of education; a lower risk in the state of São Paulo; a lower risk in peri-urban than urban areas; a higher risk in 2021 and 2022; a decrease of about 21% in the risk of die for those who received the COVID-19 vaccine (2021: 22% lower; 2022: 13% lower; 2023: no difference); a decrease of 60 to 70% in the risk of die for hospitalized individuals; and an increase of 19 to 20% in the risk of die for those who had some risk factor (Table 2).

**Table 2:** Crude and adjusted hazard ratio of COVID-19 death according to the predictors (southeast of Brazil).

	*Crude Cox model*	*Adjusted classic Cox model* ^£^	*Model 1 -Adjusted Cox mixed-effects model* ^§^	*Model 2 -Adjusted Cox mixed-effects model* ^¥^
***Hazard ratio***			*Fixed effects*	*Fixed effects*
**Age**	1.03***	1.03***	1.02***	1.03***
**Elderly**	2.14***			
**Male**	0.96***	1.07***	1.07***	1.07***
**Race**				
White	1	1	1	1
Black	1.22***	1.13***	1.11***	1.12***
Yellow	0.92***	0.93*	0.94^•^	0.92*
Brown	1.00^NS^	1.07***	1.02***	1.07***
Indian	0.74***	0.89^NS^	0.87^NS^	0.89^NS^
**Years of study**				
Zero	1	1	1	1
Five	0.83***	0.88***	0.87***	0.88***
Nine	0.69***	0.85***	0.84***	0.85***
Twelve	0.54***	0.78***	0.76***	0.78***
More than twelve	0.46***	0.63***	0.62***	0.63***
Not applicable	0.14***	0.79***	0.70***	0.70***
**State**				
Espírito Santo	1	1	1	
Minas Gerais	0.65***	0.86***	0.89^•^	
Rio de Janeiro	0.90***	1.46***	1.04^NS^	
São Paulo	0.60***	0.76***	0.75***	
**Area**				
Urban	1	1	1	1
Rural	1.22***	0.97^NS^	1.00^NS^	0.97^•^
Peri-urban	1.18***	0.87**	1.00^NS^	0.88*
**First symptoms year**				
2020	1	1	1	
2021	0.98***	1.18***	1.21***	
2022	1.23***	1.22***	1.27***	
2023	0.96**	0.97^NS^	1.07*	
**Immunization for COVID-19**	1.06***			0.79***
2021		0.78***		
2022		0.87***		
2023		1.03^NS^		
**Hospitalization**	0.32***	0.40***	0.30***	0.38***
**Risk factor**	1.40***	1.19***	1.20***	1.20***
***Random effects—SD***				
**City** (intercept)			0.35	
**State** (intercept)				4.20
**First symptoms year** (slope)				0.16
***Fit measures***				
**Likelihood ratio**		38195 (df = 21)***	5638635 (df = 1199)***	5647953 (df = 4)***
**Log-likelihood**		-1390689 (df = 21)	-1384557 (df = 937)	-1390405 (df = 20)
**AIC**		2781419	2770987	2780850
**BIC**		2781623	2780082	2781045

*** = p-value < 0.0001,

** = p-value < 0.001,

* = p-value < 0.01,

^•^ = p-value < 0.1;

SD = Standard deviation; df = degrees of freedom; AIC = Akaike Information Criterion; BIC = Bayesian Information Criterion

^£^ The classic Cox-adjusted model included the following predictors: age, sex, race, years of education, state of residence, area, year of first symptoms, hospitalization, and risk factors. To access the estimated effect of COVID-19 immunization, separately adjusted models were used for each year that COVID-19 vaccination occurred in Brazil.

^§^ The first Cox mixed-effects adjusted model included fixed effects of age, sex, race, years of study, state, area, first symptoms year, hospitalization, and risk factor; and the random effect of the municipality of residence (intercept).

^¥^ The second Cox mixed-effects adjusted model included fixed effects of age, sex, race, years of study, area, hospitalization, and risk factor; and the random effects of state (intercept) and first symptoms year (slope).

The standard deviation of the random effects of the first Cox mixed effects model was smaller than that of the second, considering that in the first there was a high number of units represented by all municipalities in the southeast region. In the second Cox mixed effects model, the number of units (intercept) was only four, representing the states of the southeast region (Table 2).

Likelihood ratio tests in the three adjusted models indicated that the inclusion of covariates was significant. The Akaike Information Criterion (AIC) and the Bayesian Information Criterion (BIC) were very close in all adjusted models, although slightly lower in the model that used municipalities as random effects and slightly higher in the classic Cox model (Table 2).

The adjusted risk of the transitioning between hospitalization and death showed few differences from the risk of death shown in Table 2: a risk 3% higher for each year of life; 8% higher in men; compared to white people, a risk of 13% higher in blacks, 7% lower in yellow, and 7% higher in brown; a decrease of the risk the higher the level of education; lowest risk in the state of São Paulo and a highest in the state of Rio de Janeiro; highest risk in urban areas; highest risk in 2021 and a lowest in 2023; a decrease of 19 to 21% and 12 to 14% in the death risk for those who received the COVID-19 vaccine in 2021 and 2022, respectively; and an increase of 26% risk for those who had some risk factor (Table 3).

**Table 3:** Crude and adjusted hazard ratio of the transitioning between individual’s conditions with COVID-19 (hospitalization and death) according to the predictors (southeast of Brazil).

	*Crude PWP model*	*Adjusted PWP model* ^£^	*Adjusted PWP fragility model* ^§^
***Hazard Ratio***			
**Age**	1.03***	1.03***	1.03***
**Elderly**	2.27***		
**Male**	0.97***	1.08***	1.08***
**Race**			
White	1	1	1
Black	1.22***	1.13***	1.13***
Yellow	0.92***	0.93**	0.93^•^
Brown	1.01*	1.07***	1.07***
Indian	0.76***	0.93^NS^	0.93^NS^
**Years of study**			
Zero	1	1	1
Five	0.85***	0.92***	0.92***
Nine	0.71***	0.90***	0.90***
Twelve	0.55***	0.83***	0.83***
More than twelve	0.46***	0.68***	0.68***
Not applicable	0.13***	0.83**	0.83**
**State**			
Espírito Santo	1	1	
Minas Gerais	0.62***	0.82***	
Rio de Janeiro	0.87***	1.42***	
São Paulo	0.58***	0.71***	
**Area**			
Urban	1	1	1
Rural	1.15***	0.93***	0.93***
Peri-urban	1.14***	0.81***	0.81***
**First symptoms year**			
2020	1	1	1
2021	0.97***	1.15***	1.15***
2022	1.17***	1.12***	1.12***
2023	0.90**	0.87***	0.87***
**Risk factor**	1.59***	1.26***	1.26***
**Immunization for COVID-19**	1.07***		
2021		0.81***	0.80***
2022		0.86***	0.88***
2023		1.04^NS^	1.01^NS^
***Gamma effect***			*(CI 95%)*
**State**			State
Espírito Santo			1.01(0.98:1.03)
Minas Gerais			0.83(0.82:0.84)
Rio de Janeiro			1.44(1.42:1.46)
São Paulo			0.71(0.71:0.72)
***Fit measures***			
**Likelihood ratio test**		77705 (df = 20)***	77705 (df = 20)***
**Log-likelihood**		-2689027 (df = 20)	-2689027 (df = 20)
**I-likelihood**			-2689050.4
**AIC**		5378095	5378095
**BIC**		5378302	5378302
**Variance of random effect**			2

*** = p-value < 0.0001,

** = p-value < 0.001,

* = p-***value*** < 0.01,

^•^ = p-value < 0.1;

df = degrees of freedom; CI = Confidence Interval; SE = standard error; PWP = Prentice, Williams & Peterson; AIC = Akaike Information Criterion; BIC = Bayesian Information Criterion

^£^ The PWP-adjusted model included the following predictors: age, sex, race, years of education, state of residence, area, year of first symptoms, and risk factors. To access the estimated effect of COVID-19 immunization, separately adjusted models were used for each year that COVID-19 vaccination occurred in Brazil.

^§^ The PWP fragility-adjusted model included the following predictors: age, sex, race, years of education, area, first symptoms year, risk factors, and the random effect of the state. To access the estimated effect of COVID-19 immunization, separately adjusted models were used for each year that COVID-19 vaccination occurred in Brazil.

The random effect variance of the state in the PWP fragility model was high. Likelihood ratio tests in the two adjusted models indicated that the inclusion of covariates was significant. Both adjusted models presented the same values for AIC and BIC.

## Discussion

Our results pointed to a decrease in the risk of dying for those who received the COVID-19 vaccine and an increase in the risk of death among different demographic groups and people with comorbidities. Men, older people, blacks, those with a lower level of education, and those living in urban areas, especially from the state of Rio de Janeiro, faced a higher risk of death.

The southeastern region of Brazil, which comprises the largest percentage of the country’s population [11], was the one that recorded the highest number of cases, hospitalizations, and deaths from COVID-19 in Brazil [12].

In this study, the highest risk of death for SARS cases classified as COVID-19 was detected in the state of Rio de Janeiro and the lowest in the state of São Paulo. Official bulletins indicate the states of Rio de Janeiro and São Paulo showed the highest overall lethality rates for COVID-19 in the Southeast (São Paulo and Rio de Janeiro– 2.69%: Minas Gerais– 1.56%: Espírito Santo– 1.11%) [12].

Despite a possible inverse relationship between temperature and mortality and incidence of COVID-19 [13], our study showed peaks in deaths mainly in summer (2021 and 2022), when temperatures are quite high. The entry of new variants into southeastern Brazil was probably the main determinant of the observed death spikes.

Our study detected a 7 to 8% increase in the risk of death in men, which is consistent with other research [14, 15]. Risk factors such as cardiovascular diseases may explain these results, as they are more prevalent among men [16]. Furthermore, men showed an especially exaggerated production of cytokines and inflammatory proteins during COVID-19, which contributes to the worsening of the disease [17]. Immune regulatory genes encoded by the X and Y chromosomes may explain lower viral loads and reduced inflammation in women than men [18]. In animal models of SARS-CoV-2 infection, differences in mortality were observed between male and female mice and were attributed to steroid hormones [19]. Multiple dimensions of biological sex, including sex steroids, sex chromosomes, and genomic and epigenetic differences between males and females, impact immune responses and may affect responses to SARS-CoV-2 infection. Consequently, some authors indicated the necessity to include sex as a biological variable in all research pipelines from fundamental research to preclinical drug development, clinical trials, and epidemiological analysis. Understanding how immunomodulatory response acts in the severity of COVID-19 could help the development of new treatments and reduce the mortality cases [20].

We detected that the risk of death in the cases practically doubled in the elderly. There was an average increase in risk of 3% for each year of life. Our results are consistent with other studies [15, 21]. In general, elderly people have several comorbidities, which reduce immune response and contribute to increasing the severity of the disease. Children are more likely to respond to viral infections than elderly patients [22]. Aging and comorbidities contribute to aggravating the COVID-19 condition, as there is an increase in pro-inflammatory mediators, causing greater susceptibility to a process of immune dysregulation after COVID-19 [22].

It is coherent to think that more vulnerable groups have a lower chance of surviving when affected by COVID-19 [23, 24]. This could explain why this study found a higher risk of death among blacks (risk about 10% higher than whites) and those with less education. COVID-19 has highlighted the social inequality in the whole world. In Brazil, vulnerable groups including dwellers in slums, the homeless, sexual and gender minorities, and indigenous have had a higher risk of SARS-CoV-2 infection and consequently, a higher risk of mortality due to issues such as lack of access to Health Units, worse socioeconomic conditions, and lower educational level [25–27].

Our study found a higher risk of death in urban areas than in rural areas, suggesting a greater spread of the disease in urban areas. This result is corroborated in other studies [28–30]. Rural geography contributed to protection against disease, considering the lower population density, which favors natural distancing and social isolation. This geography, however, may temporarily buffer the spread of the COVID-19 virus but does not prevent the effect of other factors such as poor healthcare infrastructure. An American study detected lower mortality rates in smaller urban areas, such as in the metropolitan outskirts and small or medium-sized metropolitan areas [31]. Our findings indicated a lower risk of death in peri-urban areas than in urban areas as also is described in Manaus, which belongs to the North Region of Brazil [30].

In this study, conditions considered risk factors for COVID-19 increased the risk of death by 20 to 26%. Other studies observed similar results [15]. A study in the United Kingdom reported higher death risk rates for people with Down Syndrome, transplants, sickle cell disease, HIV/AIDS, chemotherapy, dementia, Parkinson’s disease, neurological conditions, liver cirrhosis, and elderly people living in shelters [32].

Our study detected that individuals who had access to hospitalization had a lower risk of death. An Italian study found no difference in reducing mortality for patients who had access to hospitalization [33]. The different results may be related to differences in access to diagnosis, treatment, and intensive care units.

In 2021, the Technical Note of the Brazilian Immunology Society about the efficacy of vaccines for COVID-19, Oxford and Butantã, reinforces that the role of vaccination would be addressed to the reduction of symptoms of the disease but not to prevent the disease [34]. Our study only investigated the risk of death in the three months following the first symptoms of COVID-19, and found a reduction in the risk of death of around 20% and 13% in 2021 and 2022, respectively, for those who took the vaccine. Several studies corroborate these results [4, 35–37]. However, local and systemic adverse events of the COVID-19 vaccine are also highlighted in several researches [35].

### Strengths and limitations

This study was able to assess the risk of death from the date of the first symptoms until 90 days after the symptoms appeared using survival analysis. Survival analysis, with the flexibility to deal with censored data and time to event, provided a detailed view of the scenario. Adjusted models, including Classical Cox, Cox mixed effects, Prentice, Williams & Peterson (PWP), and PWP fragility models, allowed us to explore associations between several predictors and the risk of death over time.

This study focused on just the most serious cases of COVID-19, classified as Severe Acute Respiratory Syndrome, and therefore does not reflect the total magnitude of cases and deaths from COVID-19 in southeastern Brazil. The completeness of data for some notification fields that are not mandatory generates a significant loss of information. In addition to unfilled data, fields marked as “ignored” also reduce the possibility of evaluating variables.

Unfortunately, the Brazilian system for reporting adverse events from vaccines, including against COVID-19 (Post-Vaccination Adverse Events Information System (SI-EAPV)), is not open access, which makes it difficult to obtain accurate information on this issue in Brazil. However, we included references from several studies that investigated this problem in this study.

## Conclusion

This study analyzed the main determinants of the risk of death in severe cases of COVID-19 in the southeast region of Brazil, using data from the Epidemiological Surveillance Information System from 2020 to 2023. The results highlight several important findings.

A significant increase in the risk of death was observed among different demographic groups and people with comorbidities. The state of Rio de Janeiro had the highest risk of death, while São Paulo had the lowest. Men, older people, blacks, those with a lower level of education, and those living in urban areas faced a higher risk of death.

The results highlight the role of immunization, with a significant reduction in the risk of death for those vaccinated, corroborating findings from other studies. Furthermore, the analysis highlighted the influence of socioeconomic, geographic, and health factors on COVID-19 mortality.

Despite the valuable contributions of this study, it is essential to recognize its limitations, such as incomplete data and possible biases associated with notifications. However, the robust analyses and consistent findings provide a solid basis for understanding the factors that affect the risk of death from COVID-19 in the southeast region of Brazil. This information is crucial to guide public health policies, vaccination strategies, and interventions targeting higher-risk groups.

## Supporting information

S1 Data (CSV)
